# Pituitary Apoplexy Complicated by Cerebral Infarction: A Case Report

**DOI:** 10.31729/jnma.6120

**Published:** 2021-07-31

**Authors:** Biraj Pokhrel, Shambhu Khanal, Parikshit Chapagain, Gopal Sedain

**Affiliations:** 1 Maharajgunj Medical Campus, Institute of Medicine, Tribhuvan University, Kathmandu, Nepal; 2 Department of Internal Medicine, Tribhuvan University Teaching Hospital, Maharajgunj, Kathmandu, Nepal; 3 Department of Neurosurgery, Tribhuvan University Teaching Hospital, Maharajgunj, Kathmandu, Nepal

**Keywords:** *acromegaly*, *cerebral infarction*, *hemiparesis*, *pituitary apoplexy*

## Abstract

Cerebral infarction is a rare complication of pituitary apoplexy, which can result in significant morbidity if not treated on time. Pituitary apoplexy mostly occurs in pre-existing adenoma, which can remain undiagnosed until symptoms arise. Here, we present a case of a 26-year-old man with undiagnosed acromegaly who presented with left retro-orbital pain, diminished vision of the left eye, and right hemiparesis. Neuroimaging revealed large hemorrhagic sellar mass and ischemic infarction in the left middle cerebral artery territory. Emergency transcranial tumor excision was done, which resulted in significant neurological recovery.

## INTRODUCTION

Pituitary apoplexy is a neurological emergency caused by hemorrhage and/or infarction of the pituitary gland usually occurring in the pre-existing adenoma.^1^ Pituitary apoplexy is rare, incidence being two to seven percent; pituitary apoplexy causing cerebral infarction is even rarer with high morbidity and mortality.^2-4^ Only a few cases of pituitary apoplexy complicated with cerebral infarction are reported.^2^ We report a patient with undiagnosed pituitary adenoma who presented with pituitary apoplexy complicated with cerebral infarction. Emergency transcranial excision of the tumor led to marked neurological improvement.

## CASE REPORT

A 26-year-old male presented to our emergency department with progressive diminution of vision of the left eye associated with retro-orbital pain for three days. It was accompanied by sudden-onset weakness of the right upper and lower limbs along with dizziness and vomiting.

On examination, his Glasgow Coma Scale was 14/15 (Eye response E4, Verbal V4, Motor M6). Visual acuity of the left eye was reduced to light perception. He had third-degree ptosis in the left eye and right upper-motor-neuron-type facial palsy. The right upper and lower limbs had power of 1/5 (Medical Research Council muscle power scale) and demonstrated signs of upper-motor-neuron lesion: brisk reflexes and up-going plantar reflex. The left upper and lower limbs had power of 5/5.

He had acromegalic features: prominent supraorbital ridges; enlarged lips, nose, and tongue; prognathic jaw; thickened skin; and enlarged hands and feet revealed normal findings.

**Figure 1 A, B:**
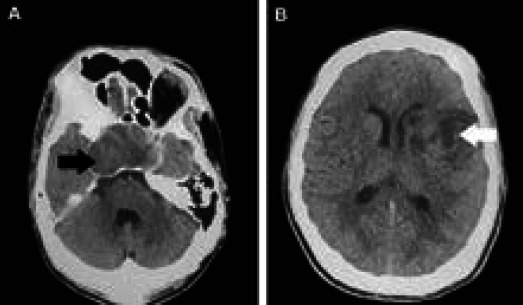
Non-contrast CT scan of the brain.

Non contrast-enhanced Computed Tomography (CT) scan of the brain revealed a sellar mass and hypodensity in the left middle cerebral artery suggesting infarction. (Figure 1).

**Figure 2 A, B:**
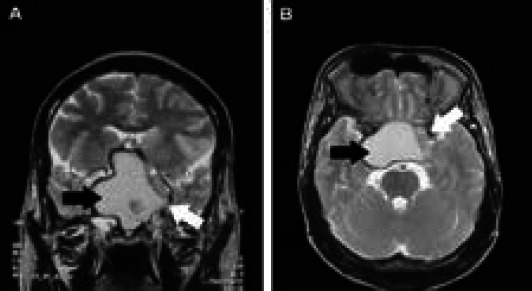
T2-weighted MRI scans of the brain.

Magnetic Resonance Imaging (MRI) of the brain revealed a large mass with hemorrhage in the sellar and suprasellar regions (shown by black arrow), which compressed the left internal carotid artery (shown by black arrow) (Figure 2).

Color Doppler of both carotid arteries showed no evidence of thrombosis or significant narrowing.

Pre-operative hormone analysis revealed high Growth hormone and Insulin-like growth factor levels. Thyroid-stimulating hormone, follicle-stimulating hormone, prolactin, free thyroxin, cortisol, and luteinizing hormone levels were normal.

Pituitary apoplexy was diagnosed based on the radiological finding of acute hemorrhage in the pituitary gland on the clinical background of acromegaly. Emergency craniotomy and excision of the tumor was done trans-cranially due to technical difficulties and the encasing of both carotids by the tumor. Intra-operatively, a well-capsulated, moderately vascular tumor with necrotic center and areas of hemorrhage was seen encasing both carotids and markedly compressing the left optic nerve. A small tuft of the tumor was attached to the sella, the optic nerve, and the carotids to the side of craniotomy. It could not be excised and was left behind. The histopathological report of the excised pituitary mass confirmed pituitary adenoma with hemorrhagic infarction.

**Figure 3 A, B:**
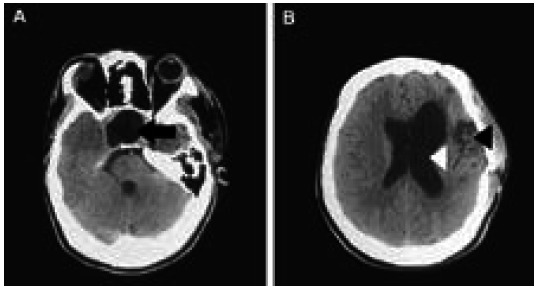
Postoperative CT scan of the brain.

CT scan of the brain done on the first postoperative day showed an empty expanded post-operative pituitary fossa (shown by black arrow) with dilated left lateral ventricle and left cortical infarct (shown by white arrow and black arrow) (Figure 3).

On the third postoperative day, he developed Diabetes Insipidus requiring desmopressin nasal spray (20mcg in 2 divided doses). Thyroid hormones and steroids were supplemented. After one week of surgery, the power of the right upper and lower limbs improved to 4 + /5. However, the vision of the left eye did not improve much, and the visual acuity remained “mild perception of light".

On follow-up after six months, he had subtle weakness in his right upper limb demonstrable as pronator drift and had power of 5/5 (Medical Research Council muscle power scale) on his right lower limb. He could walk without external support. The visual acuity of his left eye was 6/36 when assessed using Snellen's chart.

## DISCUSSION

Pituitary adenoma is one of the commonest benign tumors of the central nervous system. It can be classified as macroadenoma (≥1cm) or microadenoma (<1cm) based on its size and as functional or nonfunctional tumor based on its ability to produce hormone. Pituitary apoplexy mostly occurs in nonfunctional adenomas, only a few studies report its occurrence in functional tumors.^5-7^ Our case had evidence of a functional pituitary tumor: acromegalic features and high growth hormone.

The most common predisposing factor for pituitary apoplexy is hypertension.^5^ Other predisposing factors include anti-coagulants, oral contraceptive pills, head trauma, acromegaly, Cushing's syndrome, aspirin therapy, and major surgeries.^5,6,8,9^ In our case, the unrecognized acromegaly might have predisposed to apoplexy.

Cerebral infarction attributable to pituitary apoplexy is an uncommon event presenting most commonly with headache, hemiparesis and loss of consciousness.^4,5^ Headache, which may be due to dural traction or meningeal irritation, is sudden and severe and can be retro-orbital, bifrontal, or sub-occipital.^10^ Cranial nerves II, III, IV, and VI can get compressed, causing vision deficits and oculomotor palsies.^4,5,10^ Cerebral infarction is thought to be caused by mechanical compression of the intracranial vessels, or cerebral vasospasm, or both, with studies showing vessel compression to be the more common mechanism.^2,11,12^ Vasospasm may be induced by subarachnoid hemorrhage, the release of vasoactive substances from the adenoma, or the release of spasmogenic factors from hypothalamic damage.^11^

In patients with pituitary apoplexy, the rapid expansion of intrasellar contents can markedly raise intrasellar pressure. Normal intrasellar pressure is believed to be similar to or less than the normal intracranial pressure of 7-15mm Hg.^13^ Intrasellar pressure can rise as high as 58 mm Hg in pituitary apoplexy.^14^ Elevated pressure causes the tumor to invade the cavernous sinus, narrowing or occluding the internal carotid artery.^14^ Occlusion is usually unilateral, and the supraclinoid and cavernous segment of the ICA are most commonly affected.^12^

The primary goal of treatment is to reduce the intra-sellar pressure and to restore the blood flow. Both surgical and conservative management are described, but there is no strong evidence for recommending one over another due to the lack of randomized controlled trials assessing outcomes in the two groups.^4,15^ Surgical decompression is preferred over conservative management if the patients have severe and progressive neuro-ophthalmological deficits.^15-6^ Early decompression surgery may reverse visual compromise and restore blood flow in the internal carotid artery.^17^ On the contrary, Clark, et al. suggest that early removal of the pituitary tumor increases the risk of hemorrhagic transformation following the recanalization of the obstructed arteries, and they recommend delayed decompression following conservative therapy with steroids as the better treatment strategy.^18^

Surgery is usually done via the trans-sphenoidal approach, but if the tumor has invaded the cavernous sinus, the transcranial approach is preferred, which confers the benefits of direct exposure of the lesion from surgical point of view and the absence of hindrance from neurovascular structures.^19^

In our case, we decided to perform emergency surgery due to the presence of hemiparesis and visual deficit. We selected the transcranial approach due to technical difficulties and due to encasing of both carotid arteries by the tumor.

The post-operative period is crucial. Endocrine abnormalities like transient diabetes insipidus and decreased production of thyroid hormones and steroids can develop, so endocrine monitoring is needed.^5,20^ Patients may need life-long hormone replacement therapy because the pituitary function may not recover completely after the surgery.^5-14^ In our case, the patient developed diabetes insipidus and hypothyroidism postoperatively. Diabetes insipidus corrected eventually but had to put on long-term thyroid hormone and steroid therapy. The neurological status improved significantly after the surgery. Cerebral infarction is a rare complication of pituitary apoplexy, and surgical versus conservative management of such cases is debatable. This study supports the recommendation that early emergency neurosurgery should be performed in pituitary apoplexy with cerebral infarction if there are significant visual and neurological deficits as it can markedly improve the neurological outcome.
